# Proteomic Trajectories of Metabolic and Proteostatic Adaptation During Normothermic Liver Perfusion

**DOI:** 10.1111/liv.70629

**Published:** 2026-04-14

**Authors:** Heithem Jeddou, Anne‐Aurélie Raymond, Jean‐William Dupuy, Cyril Dourthe, Hector Prudhomme, Eya Ben Nejma, Stylianos Tzedakis, Bruno Turlin, Pierre Allaume, Karim Boudjema, Michel Samson

**Affiliations:** ^1^ Service de Chirurgie Hépatobiliaire et Digestive, CHU Rennes Rennes France; ^2^ Inserm, EHESP Université de Rennes, Irset (Institut de Recherche en Santé, Environnement et Travail) – UMR_S 1085 Rennes France; ^3^ Université de Bordeaux, CNRS, INSERM, TBM‐Core, US5, UAR 3427 Bordeaux France; ^4^ Service de Chirurgie Digestive, Hépatobiliaire et Endocrinienne, Hôpital Cochin, AP‐HP Université Paris Cité Paris France; ^5^ Service D'anatomie Pathologique, CHU Rennes Rennes France

**Keywords:** biliary complications, graft viability, ischemia–reperfusion injury, liver transplantation, normothermic machine perfusion, proteomics

## Abstract

**Background:**

Normothermic machine perfusion (NMP) enables metabolic restoration and viability testing of liver grafts, but current viability criteria incompletely predict post‐transplant outcomes. The molecular basis of graft resilience or biliary vulnerability remains unclear. This study aimed to characterise tissue‐level proteomic trajectories during NMP and early reperfusion to identify molecular signatures associated with biliary complications after liver transplantation (LT).

**Methods:**

This prospective, single‐centre study was conducted at Rennes University Hospital. Twenty donation‐after‐brain‐death (DBD) livers underwent NMP; sixteen transplanted grafts with complete sequential biopsies and ≥ 6 months of follow‐up were analysed. Biopsies were collected after cold storage (B1), at the end of NMP (B2), and 1 h after graft reperfusion (B3). Proteins were quantified by high‐resolution LC–MS/MS and analysed with Proteome Discoverer 3.1/Chimerys. Pathway enrichment used Ingenuity Pathway Analysis to compare grafts with and without biliary complications.

**Results:**

Principal component analysis revealed distinct proteomic profiles between grafts with and without complications at all biopsy time points. During NMP (B2/B1), uncomplicated grafts showed glycolytic activation with attenuation of oxidative phosphorylation, whereas complicated grafts showed blunted glycolysis and mild OXPHOS upregulation. At reperfusion (B3/B2), complicated grafts displayed induction of translational and endoplasmic‐reticulum‐stress pathways, while resilient grafts maintained proteasome‐related protein turnover and enrichment of a hypoxia‐response signature driven by ELOC and proteasome subunits.

**Conclusions:**

Sequential tissue proteomics during NMP reveals divergent metabolic and proteostatic adaptations linked to biliary outcomes. Glycolytic activation with preserved protein turnover characterises resilient grafts, whereas translational and ER‐stress programmes predominate in complicated ones. These insights may refine viability assessment beyond biochemical criteria.

## Summary

Some liver grafts develop biliary complications after transplantation despite meeting current viability criteria during machine perfusion. By analysing protein changes in liver tissue during normothermic perfusion and early reperfusion, we identified distinct metabolic and stress‐response patterns associated with subsequent biliary outcomes. These findings may help refine future assessment and preservation strategies for donor livers.

**Abbreviations**
CIT: cold ischemia time
DBD: donation‐after‐brain‐death
DRI: donor risk index
EAD: early graft dysfunction
ECD: extended criteria donors
ERCP: endoscopic retrograde cholangiopancreatography
IPA: Ingenuity Pathway Analysis
IRI: ischemia–reperfusion injury
LFQ: label‐free quantification
LT: liver transplantation
MELD: Model for End‐Stage Liver Disease
MRCP: magnetic resonance cholangiopancreatography
NAS: non‐anastomotic strictures
NMP: Normothermic machine perfusion
SCS: static cold storage

## 1 Introduction

Liver transplantation (LT) is the only curative treatment option for patients with end‐stage liver disease. However, a persistent disparity remains between supply and demand for donor organs suitable for transplantation, resulting in waiting list mortality rates of up to 20% [1]. This shortage has led to an increasing use of high‐risk grafts from extended criteria donors (ECD). These ECD grafts include livers from donation after circulatory death (DCD), as well as livers from donors with risk factors such as advanced age or comorbidities including diabetes mellitus or severe macrosteatosis [2]. Static cold storage (SCS) remains the gold standard for liver graft preservation prior to transplantation. Although effective for optimal grafts, SCS provides limited protection for marginal organs and precludes functional assessment [3]. Biliary complications remain a major source of morbidity after LT, particularly in grafts exposed to prolonged ischemia or recovered from DCD donors. Ischemic cholangiopathy, anastomotic and non‐anastomotic strictures impair graft function and may ultimately lead to graft loss or re‐transplantation. Given that biliary outcomes are central determinants of long‐term graft survival, improved tools to predict and mechanistically understand biliary injury are critically needed [4, 5, 6]. Normothermic machine perfusion (NMP) has therefore emerged as a physiologic preservation strategy that restores cellular metabolism and mitigates ischemic injury, particularly in high‐risk grafts [7, 8]. By maintaining the liver at physiological temperature (35°C–37°C) under oxygenated perfusion, NMP also enables real‐time evaluation of graft viability [9, 10, 11, 12, 13, 14, 15, 16].

Functional assessment during NMP currently relies on surrogate biochemical markers of hepatocellular and cholangiocellular function [10, 11, 17, 18]. Hepatocellular viability is evaluated through lactate clearance, bile production, and transaminase release [9, 11, 19, 20, 21], whereas biliary viability is increasingly assessed via bile composition (pH, bicarbonate and glucose) [9, 11, 22, 23, 24]. Combining these criteria improves graft selection and reduces post‐transplant complications, particularly ischemic cholangiopathy [17, 18, 22, 23, 24].

However, these criteria remain empirical and capture only a fraction of the liver's adaptive capacity [18, 20]. Some grafts that meet accepted thresholds may still fail, while others are unnecessarily discarded [8, 16]. The mechanisms determining biliary vulnerability or resilience remain poorly understood [11]. Although preservation of peribiliary glands and microvasculature has been associated with bile composition and regenerative potential, the underlying molecular mechanisms remain poorly understood, underscoring the need for deeper mechanistic insight [9, 11, 20].

High‐throughput molecular technologies now allow exploration of these mechanisms beyond conventional biochemistry [25]. Moreover, emerging methodological approaches, either real‐time spectral tissue profiling (Mass spec Pen or Spider Mass) [26, 27] or combining tissue proteome analysis by LC–MS/MS with machine learning to assign a patient to a reference group [28] are promising for clinical implementation to address unmet clinical needs.

Similar strategies integrating proteomic profiling with machine learning approaches have already been applied in liver diseases to identify diagnostic biomarkers and stratify disease states, including nonalcoholic fatty liver disease and hepatocellular carcinoma [29, 30]. Discovery proteomic approaches already enable identification of pathways involved in energy metabolism, stress response, inflammation, and tissue repair during preservation and reperfusion [31]. Previous bile proteomic studies during NMP revealed signatures of biliary regeneration and candidate biomarkers of graft viability [32]. Yet, proteomic characterisation of liver tissue itself has been scarcely investigated, and no study has systematically linked dynamic tissue proteomic changes to post‐transplant biliary outcomes.

Building on this rationale, we performed sequential tissue proteomic analysis of liver biopsies obtained before, during and after NMP. These time points were selected to capture key phases of preservation and reperfusion: cold ischemia, metabolic adaptation during NMP and early reperfusion injury. Our objective was to delineate adaptive versus maladaptive proteomic trajectories during preservation and reperfusion, and to identify molecular pathways associated with metabolic recovery, proteostatic control, and postoperative biliary outcomes by systematically correlating dynamic liver tissue proteomics during NMP with post‐transplant biliary complications.

## 2 Materials and Methods

### 2.1 Study Design and Ethical Approval

This prospective, observational study was conducted at Rennes University Hospital (France) in collaboration with IRSET (INSERM UMR 1085, Rennes) and the OncoProt proteomics platform (Bordeaux, France). Liver grafts were preserved using end‐ischemic NMP within the framework of the OVERNIGHT protocol, an institutional clinical programme developed at our centre, designed to extend preservation time and facilitate daytime transplantation. The present proteomic investigation was conducted as an ancillary translational study within this programme.

Between March 2021 and July 2023, all adult patients undergoing liver‐only transplantation were screened for eligibility. Inclusion required being the recipient of a DBD graft in which hepatectomy was completed between 16:00 and 00:00, thereby qualifying the liver for OVERNIGHT NMP. According to this protocol, perfusion was initiated upon graft arrival and maintained until the following morning, when transplantation was performed during regular working hours.

Although the initial purpose of the OVERNIGHT protocol was logistical optimisation, graft viability was systematically assessed during NMP according to predefined criteria [20]. Liver biopsies were collected at standardised time points for ancillary translational analyses.

The study protocol was approved by the institutional ethics committee (University Hospital Rennes, Approval No. 25.139) and complied with French regulations for research involving human biological material (MR‐004). Written informed consent for the use of donor tissue and recipient clinical data was obtained in accordance with national guidelines.

### 2.2 Donor Grafts and Perfusion Protocol

All liver grafts were procured by our local multiorgan procurement team using standard techniques as detailed in Supporting Information: methods. Following procurement, livers were transported in SCS to our centre and subsequently prepared for NMP.

Perfusion was performed using the OrganOx Metra system under continuous dual perfusion at a target temperature of 37°C. Typical target flows were 200–400 mL/min through the hepatic artery and 1000–1200 mL/min through the portal vein, with inferior vena cava pressure maintained between 0 and 2 mmHg. The perfusate consisted of 500 mL Gelofusine supplemented with three units of donor‐matched packed red blood cells, insulin, heparin, prostacyclin, calcium gluconate, magnesium sulphate, sodium bicarbonate (titrated to maintain pH 7.35–7.45), antibiotics and parenteral nutrition (Nutriflex), administered according to institutional standard dosing protocols.

Perfusate blood gas analyses (including lactate and glucose) were performed hourly in the central biochemistry laboratory. In parallel, perfusate pH, oxygenation, hemodynamic parameters (arterial and portal flows and pressures) and bile production were continuously recorded by the device. Detailed procurement procedures and perfusion settings are provided in the Supporting Information.

### 2.3 Viability Assessment

Graft viability was assessed at 240 min according to predefined criteria (Table S1), as previously described [19].

For a graft to be considered viable and eligible for transplantation, a perfusate lactate level ≤ 2.5 mmol/L was mandatory, together with fulfilment of at least two additional criteria among the following: sustained bile production, perfusate pH ≥ 7.30, evidence of glucose consumption, stable hemodynamic flows (arterial ≥ 150 mL/min and portal ≥ 500 mL/min), and a homogeneous perfusion pattern with uniform macroscopic graft appearance [10, 19]. Grafts meeting all viability criteria were considered suitable for transplantation. Only those transplanted grafts with complete sequential biopsies and postoperative follow‐up were included in the present analysis.

### 2.4 Tissue Sampling

Three sequential wedge biopsies were obtained from each transplanted graft:

- B1: immediately after cold storage (pre‐NMP);
- B2: at the end of NMP;
- B3: one hour after graft reperfusion.

Biopsies were snap‐frozen in liquid nitrogen and stored at −80°C until analysis.

A total of 48 fresh‐frozen biopsies (16 grafts × 3 time points) were processed for proteomic analysis using high‐resolution LC–MS/MS (Orbitrap Exploris 480) in data‐dependent acquisition mode.

Additional details regarding procurement, perfusion settings, and post‐transplant management are described in Supporting Information: Methods.

### 2.5 Clinical Outcomes Definition

For proteomic correlation, only transplanted grafts with complete sequential biopsies and ≥ 6 months of follow‐up were included (*n* = 16).

Biliary complications were defined as anastomotic or non‐anastomotic strictures and bile leaks. Strictures were confirmed by magnetic resonance cholangiopancreatography (MRCP). Bile leaks were diagnosed based on bilious output in surgical drains and/or radiological evidence of leakage on contrast‐enhanced CT imaging. These complications were managed according to institutional protocols (details provided in Table S3).

### 2.6 Proteomic Sample Preparation for Mass Spectrometry

Tissue was lysed in 100 mM Tris buffer supplemented with 1% SDS and protease inhibitors (Complete, Roche). Mechanical lysis was performed with tungsten beads in a tissue homogeniser, followed by ultrasonication. Lysates were cleared by centrifugation at 10 000 g for 15 min at 4°C, and protein concentration was measured by BCA assay (Pierce, Thermo Scientific).

Proteins were reduced with 30 mM dithiothreitol (DTT, 56°C, 1 h) and alkylated with 90 mM iodoacetamide (1 h, room temperature, dark). Proteins were digested using the single‐pot, solid‐phase‐enhanced sample preparation (SP3) method [29] on a Biomek i5 automated workstation (Beckman Coulter Life Sciences). Peptides were quantified using a fluorometric peptide assay (Pierce, Thermo Scientific).

### 2.7 Mass Spectrometry Analysis

NanoLC‐MS/MS analysis were performed using a Vanquish Neo UHPLC System (Thermo Scientific) associated to Orbitrap Exploris 480. The peptide extracts (200 ng) were loaded onto a 5 mm × 300 μm ID PepMap Neo Trap Cartridge (C18, 5 μm particle size, 100 Å pore size, Thermo Scientific) and separated on an analytical column (25 cm × 75 μm ID, 1.7 μm, C18 beads, Ionopticks) at a flow rate of 300 nL/min at 50°C using a multistep gradient of 3%–25% mobile phase B (80% MeCN in 0.1% formic acid) for 45 min and 25%–35% B for 15 min, 35%–95% B for 1 min and an 11‐min wash at 99% B. The mass spectrometer operated in positive ion mode at a 1.4 kV needle voltage, and data were acquired using Xcalibur 4.5 software in a data‐dependent mode. MS scans (m/z 375–1500) were recorded at a resolution of *R* = 120 000 (@m/z 200), a standard AGC target, and an injection time in automatic mode, followed by a top speed duty cycle of up to 1 s for MS/MS acquisition. Precursor ions (2–6 charge states) were isolated in the quadrupole with a mass window of 2 Th and fragmented with HCD@30% normalised collision energy. MS/MS data were acquired with a resolution of *R* = 15 000 (@m/z 200), a standard AGC target, and a maximum injection time in automatic mode. Selected precursors were excluded for 45 s.

### 2.8 MS Data Analysis

Protein identification and label‐free quantification (LFQ) were done in Proteome Discoverer 3.1. The CHIMERYS node using the prediction model inferys_3.0.0 fragmentation was used to identify proteins in batch mode by searching against the UniProt *Homo sapiens* database (82 233 entries, released June 2024). Two missed enzyme cleavages were allowed for trypsin. Peptide lengths of 7–30 amino acids, a maximum of 3 modifications, charges of 2–4, and 20 ppm for fragment mass tolerance were set. Oxidation (M) and carbamidomethyl (C) were respectively searched as dynamic and static modifications by the CHIMERYS software. Peptide validation was performed using the Percolator algorithm and only “high confidence” peptides were retained corresponding to a 1% false discovery rate at the peptide level. Minora feature detector node (LFQ) was used along with the feature mapper and precursor ions quantifier. The normalisation parameters were selected as follows: (1) Unique peptides, (2) Precursor abundance based on intensity, (3) Normalisation mode: total peptide amount, (4) Protein abundance calculation: summed abundances, (5) Protein ratio calculation: pairwise ratio based (protein ratios are calculated as the median of all possible pairwise peptide ratios calculated between replicates of all connected peptides) and (6). Quantitative data were considered for master proteins, quantified by a minimum of 2 unique peptides, a fold change above 2 and a statistical *p*‐value adjusted using Benjamini–Hochberg correction for the FDR lower than 0.05. The mass spectrometry proteomics data have been deposited to the ProteomeXchange Consortium via the PRIDE [33] partner repository with the dataset identifier PXD067270.

Protein expression ratios were calculated for each patient between the different time points (B2/B1, B3/B2 and B3/B1) by taking the median of the abundances of the unique peptides identified for each protein. We used the Low Abundance Resampling (LAR) method in Proteome Discoverer 3.2 to impute missing peptide values with low estimates (missing values were replaced with random values sampled from the lower 5% of detected values), allowing accurate distinction between true absent/present signals and undetected low‐abundance peptides, thereby preserving quantitative consistency for downstream analyses.

Statistical comparisons were performed using nonparametric Wilcoxon–Mann–Whitney *t*‐tests to identify proteins differentially expressed between grafts with and without post‐transplant complications.

Principal component analysis (PCA) and hierarchical clustering heat maps were generated after reducing the dataset to significant proteins to visualise sample clustering and group separation. Functional enrichment and pathway analysis were performed using Ingenuity Pathway Analysis (IPA, Qiagen) to identify biological processes associated with post‐perfusion and post‐reperfusion changes and to explore differences according to clinical outcome.

## 3 Results

### 3.1 Study Cohort

Between March 2021 and July 2023, 20 liver grafts underwent preservation according to the OVERNIGHT protocol. Of these, 18 were transplanted and 2 were discarded during NMP due to inadequate lactate clearance. Individual viability parameters of perfused grafts are summarised in Table S2. Two recipients died early postoperatively (one from severe pancreatitis and one from arterial thrombosis requiring re‐transplantation), precluding assessment of biliary outcomes. Accordingly, the final proteomic analysis included 16 transplanted grafts with complete sequential biopsy sampling and at least 6 months of follow‐up. A minimum follow‐up of 6 months was selected to ensure sufficient follow‐up time for the development and detection of postoperative biliary complications [34].

### 3.2 Donor and Recipient Characteristics

Baseline donor characteristics according to the occurrence of biliary complications are shown in Table 1. All grafts were obtained from DBD donors. Median donor age, donor risk index (DRI) and cold ischemia time (CIT) did not differ significantly between groups. Specifically, median donor age was 70 years (56–75) in grafts that later developed biliary complications versus 68 years (55–74) in those that did not. The median DRI was 1.72 (1.54–1.93) and 1.70 (1.50–1.94), and median CIT was 328 min (295–375) and 325 min (293–372), respectively. Other donor‐related variables, including cause of death, serum transaminase levels and serum sodium concentration, were likewise not significantly different between groups. Recipient characteristics are presented in Table 2. Median recipient age was 60 years (51–63) in the biliary complication group and 59 years (52–62) in the group without complications. Model for End‐Stage Liver Disease (MELD) scores, sex distribution and body mass index were likewise not significantly different between groups. With a median follow‐up of 24 months (12–39), both graft and patient survival were 100% in each group.

**TABLE 1:** Donor characteristics for included livers.

	Biliary complications (*n* = 7)	No biliary complications (*n* = 9)	*p*
Age (years)	70 (56–75)	68 (55–74)	0.74
BMI (kg/m^2^)	32 (25–34)	31 (25–33)	0.68
Gender			
Male	5 (71)	7 (78)	1.00
Female	2 (29)	2 (22)	
Cause of death			
Trauma	2 (29)	3 (33)	0.88
CVA	4 (57)	4 (44)	
Anoxia	1 (14)	2 (22)	
Donor type			
DBD	7 (100)	9 (100)	
DCD	0 (0)	0 (0)	
Cardiac arrest prior to organ procurement	1 (14)	0 (0)	0.44
Last sodium > 155 mmol/L	2 (29)	2 (22)	1.00
Last AST > 150 U/L	1 (14)	0 (0)	0.44
Last ALT > 170 U/L	1 (14)	1 (11)	1.00
IUC > 7 days	0 (0)	0 (0)	1.00
ECD	6 (86)	7 (78)	1.00
SCI time (min)	328 (295–375)	325 (293–372)	0.81
NMP time	720 (300–965)	710 (290–968)	0.89
DRI	1.72 (1.54–1.93)	1.70 (1.50–1.94)	0.77

*Note:* Values are expressed as median (IQR) or *n* (%). Continuous variables were compared using the Mann–Whitney U test and categorical variables using Fisher's exact test.

Abbreviations: ALT, alanine aminotransferase; AST, aspartate aminotransferase; BMI, body mass index; CVA, cerebrovascular accident; DBD, donation after brain death; DCD, donation after circulatory death; DRI, donor risk index; ECD, extended criteria donor; IUC, intensive care unit stay; NMP, normothermic machine perfusion; SCI, static cold ischemia.

**TABLE 2:** Recipient characteristics by biliary outcome.

	Biliary complications (*n* = 7)	No biliary complications (*n* = 9)	p
Age (years)	60 (51–63)	59 (52–62)	0.84
Gender			
Male	5 (71)	7 (78)	1.00
Female	2 (29)	2 (22)	
BMI (kg/m^2^)	32 (25–34)	31 (25–33)	0.72
MELD score	22 (17–31)	23 (17–32)	0.91
*Post‐transplant outcomes*			
Graft survival			
3 months	7 (100)	9 (100)	—
12 months	7 (100)	9 (100)	—
24 months	7 (100)	9 (100)	—
Recipient survival			
12 months	7 (100)	9 (100)	—
24 months	7 (100)	9 (100)	—
Peak ALT (U/L)	540 (72–3000)	530 (80–3050)	0.88
Peak AST (U/L)	515 (130–4100)	505 (120–4200)	0.93
Bilirubin day 7 (μmol/L)	66 (10–220)	64 (7–218)	0.81
INR day 7	1.13 (1.02–1.61)	1.14 (1.03–1.62)	0.79
GGT (U/L)			
Day 30	220 (35–780)	210 (33–790)	0.87
Day 90	75 (30–540)	70 (27–530)	0.82
Day 180	37 (21–400)	35 (20–405)	0.89
ALP (U/L)			
Day 30	135 (70–470)	128 (67–460)	0.84
Day 90	82 (60–410)	78 (57–405)	0.86
Day 180	112 (75–490)	110 (70–495)	0.90
Primary non‐function	0 (0)	0 (0)	—
Hepatic artery thrombosis	0 (0)	0 (0)	—
Acute rejection	0 (0)	0 (0)	—
Chronic rejection	0 (0)	0 (0)	—
Reoperation for bleeding	0 (0)	0 (0)	—

*Note:* Values are expressed as median (IQR) or *n* (%). Continuous variables were compared using the Mann–Whitney U test and categorical variables using Fisher's exact test.

Abbreviations: ALP, alkaline phosphatase; ALT, alanine aminotransferase; AST, aspartate aminotransferase; BMI, body mass index; GGT, gamma‐glutamyl transferase; INR, international normalised ratio; MELD, Model for End‐Stage Liver Disease.

### 3.3 Biliary Complications

Biliary complications occurred in 7 of 16 recipients (43%), including 2 non‐anastomotic strictures, 3 anastomotic strictures (Figure S1), and 2 bile leaks. Strictures were confirmed by MRCP, whereas bile leaks were diagnosed based on postoperative imaging or the presence of bile in surgical drains. Management included ERCP with stenting, reoperation or Roux‐en‐Y hepaticojejunostomy, according to standard institutional protocols (Table S3).

### 3.4 Functional Characteristics During NMP

**FIGURE 1:**
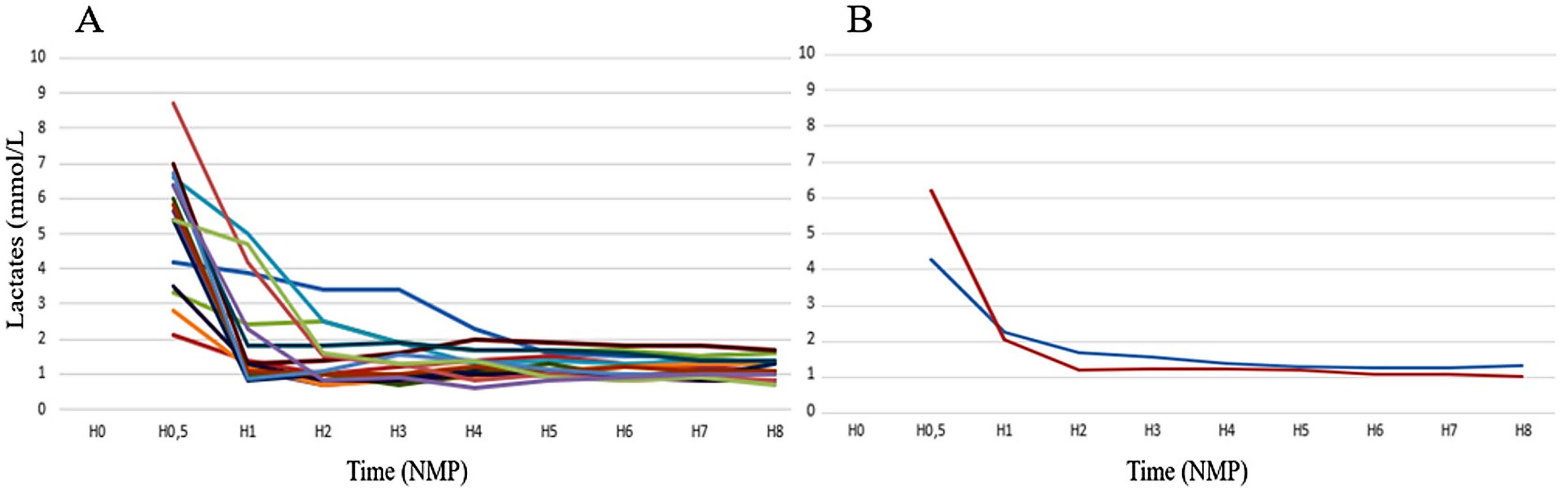
Evolution of lactate levels during normothermic machine perfusion in 16 analysed grafts. (blue: No biliary complications; red: Biliary complications).

Functional assessment during NMP showed no apparent differences between grafts that subsequently developed biliary complications and those that did not. Perfusate lactate clearance, measured in the central laboratory, and device‐monitored parameters, including pH, oxygenation, glucose consumption, arterial and portal flows, and bile output, showed overlapping distributions between groups (Figure 1, Table S2). Given the limited sample size and the exploratory nature of the study, these comparisons should be interpreted cautiously as the analysis was not powered to detect minor differences.

### 3.5 Kinetics of Graft Evolution by Proteomic Discovery Approach

#### 3.5.1 Distinct Proteomic Profiles at Different Biopsy Time Points According to Clinical Groups

**FIGURE 2:**
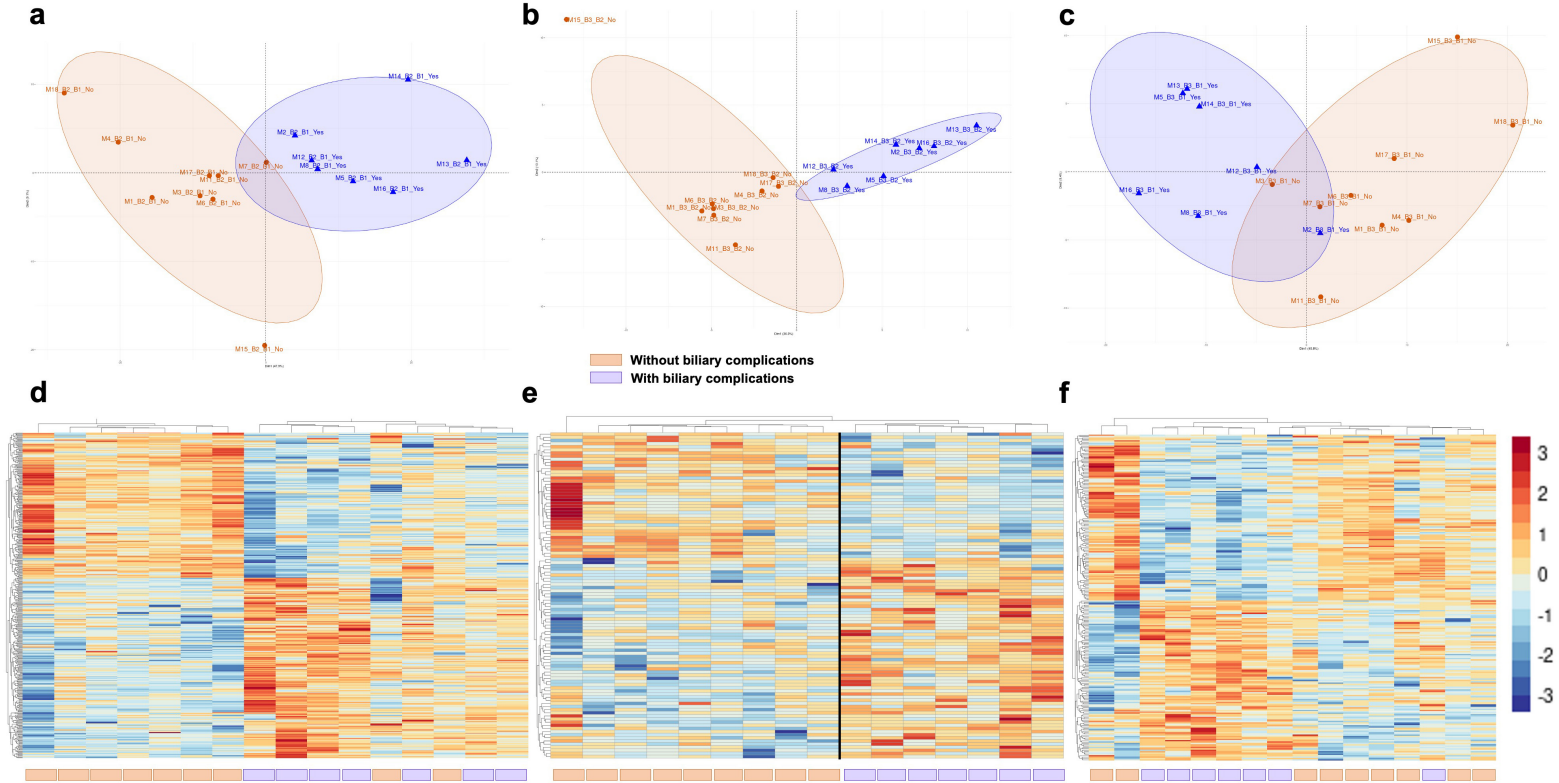
Comparison of proteomic profiles between grafts with and without biliary complications. (a) PCA of protein abundance ratios between biopsies B2 and B1 (end vs. start of NMP); (b) PCA of protein abundance ratios between B3 and B2 (reperfusion vs. end of NMP); (c) PCA of protein abundance ratios between B3 and B1 (reperfusion vs. pre‐NMP); (d–f) hierarchical clustering heatmaps of significantly regulated proteins (dataset filtered using a Mann–Whitney test, *p* < 0.05) for each respective comparison, presented according to biliary complication status. Proteins are displayed on the *y*‐axis and samples on the *x*‐axis.

Principal Component Analysis (PCA) of relative protein abundance ratios revealed a clear separation between grafts with and without biliary complications at all biopsy comparison time points. Distinct proteomic profiles were observable early between B2 and B1 (Figure 2a, Table S4), reflecting changes induced solely by normothermic perfusion. The discrimination between the two clinical groups was complete when comparing B3 and B2, highlighting differential modifications occurring between the end of perfusion and transplantation (Figure 2b, Table S4). The separation between B3 and B1, representing the overall changes in the graft throughout the entire management process, was less pronounced (Figure 2c, Table S4). Hierarchical clustering of significantly regulated proteins (*p* < 0.05), visualised as heatmaps (Figure 2d–f), confirmed these findings and allowed visualisation of distinct proteomic expression profiles between clinical groups.

### 3.6 A Reactive Energy Metabolism in Uncomplicated Grafts

**FIGURE 3:**
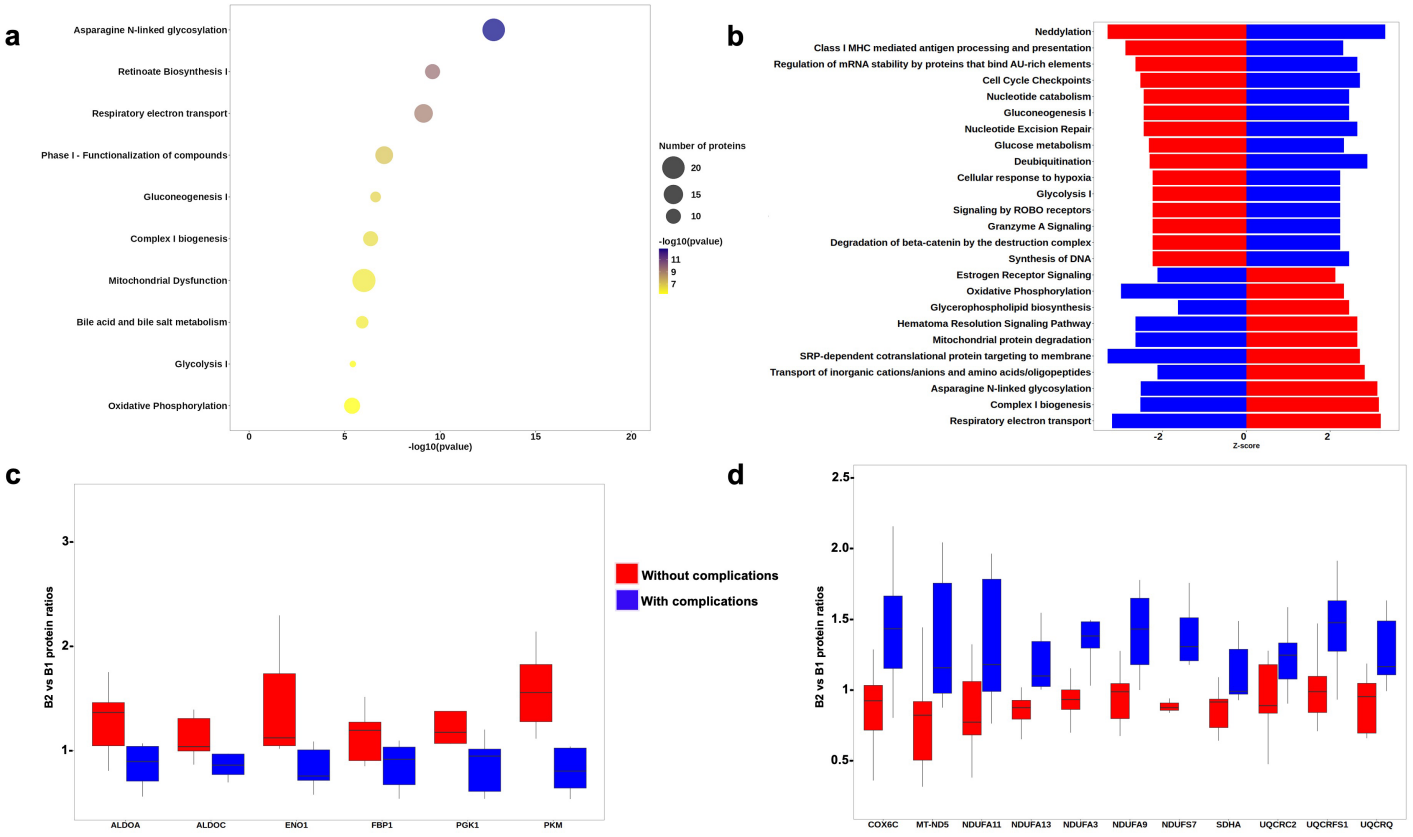
Metabolic pathway analysis during normothermic perfusion. (a) Pathway enrichment analysis using the IPA database of B2 vs. B1 showing pathways associated with energy and bile acid metabolism according to graft group; (b) *Z*‐score representation of pathways exhibiting opposite regulation patterns between groups; (c) relative abundance of glycolysis‐associated proteins in B2 vs. B1, presented by graft group; (d) relative abundance of oxidative phosphorylation–associated proteins in B2 vs. B1, presented by graft group.

Pathway's enrichment analysis was performed using the Ingenuity Pathway Analysis platform (IPA, Qiagen) (Table S5) identified significant enrichment of energy metabolism (30 proteins related) and bile acid metabolism (8 proteins related) pathways in grafts showing early divergence between those that did and did not develop biliary complications (B2 vs. B1; Figure 3a). *Z*‐score analysis revealed inverse regulation of several biological pathways between the two clinical groups (Figure 3b). Notably, a metabolic shift characterised by increased glycolytic activity and decreased mitochondrial respiration (oxidative phosphorylation) was observed in non‐complicated grafts, whereas the opposite profile was evident in grafts that developed complications (Figure 3b).

Proteomic analysis of energy metabolism pathways further confirmed a marked upregulation of glycolytic proteins in non‐complicated grafts, which was absent in complicated ones (Figure 3c). In contrast, a modest increase in oxidative phosphorylation‐related proteins was observed in grafts associated with complications (Figure 3d). These findings suggest an early, differential metabolic reprogramming during normothermic perfusion that may influence subsequent graft outcomes.

### 3.7 Reactive Mechanisms Operating on Different Timescales in Grafts With Complications

**FIGURE 4:**
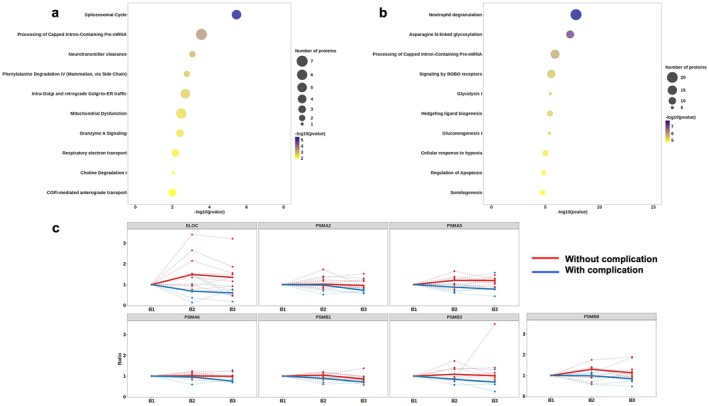
Temporal analysis of stress and proteostasis‐related pathways during reperfusion. (a) IPA pathway enrichment analysis for the B3 vs. B2 and B3 vs. B1 comparisons, displaying translation‐related proteins according to graft group; (b) IPA pathway enrichment analysis for B3 vs. B1, presenting metabolic‐ and stress‐response–related pathways by graft group; (c) differential expression of proteins included in the hypoxia‐response pathway (e.g., ELOC and proteasome subunits) in the B3 vs. B1 comparison, presented according to graft group. Bold curves represent the median value across all patients within each group.

We also performed a pathway enrichment analysis using the IPA database on datasets comparing B3 vs. B2 and B3 vs. B1 (Figure 4a, Table S5). We observed the same energy imbalance between the two clinical groups. However, the other biological pathways that were differentially regulated between the perfusion and post‐transplantation phases differed. Most of the proteins that were significantly differentially expressed between the two clinical groups were associated with protein translation (spliceosomal cycle, processing of capped intron‐containing pre‐mRNA, intra‐Golgi and retrograde Golgi‐to‐ER trafficking with 12 proteins related), with an overexpression of these proteins in grafts that developed biliary complications. These results suggest that grafts with biliary complications undergo a reactive reprogramming of protein translation in the tissue. However, the time required for major protein synthesis and the actual duration of the transplantation process are not compatible.

When performing a global pathway enrichment analysis of the proteomic profile evolution between B1 and B3, the main previously identified biological pathways reemerge (glycolytic shift and reactive expression reprogramming) (Figure 4b). Additionally, we observe significantly enriched biological pathways related to stress response and inflammation (neutrophil degradation, regulation of apoptosis with 24 proteins related) (Figure 4b). Interestingly, the cellular response to hypoxia pathway is significantly different between the two clinical groups. Upon closer examination of the proteins contributing to the enrichment of this pathway (Figure 4c, Table S5), we identified ELOC and several proteasome subunits (PSMA2, PSMA5, PSMA6, PSMB1, PSMB3 and PSMB9), which have been associated with hypoxia resistance and are slightly but significantly overexpressed in grafts without complications [35]. Although caution is warranted regarding this association with hypoxia, as we did not identify canonical proteins such as HIF, PHD or VHL.

Altogether, these results suggest that grafts without complications may have a better capacity to maintain tissue viability through a more efficient and rapid stress response, with also an adaptation of energy supply that likely contributes to this support.

## 4 Discussion

This study provides the first systematic, tissue‐resolved proteomic characterisation of human liver grafts during clinical NMP and early reperfusion. By profiling sequential parenchymal biopsies and linking molecular signatures to post‐transplant outcomes, we identified two divergent adaptive trajectories. Grafts without biliary complications displayed early glycolytic activation and preserved protein turnover, whereas those that later developed complications showed blunted glycolysis, maladaptive translational reprogramming and sustained stress responses. These findings refine current understanding of graft adaptation to machine perfusion and reperfusion and highlight the potential of tissue proteomics to inform mechanism‐based viability evaluation. Currently, graft viability evaluation during NMP relies on pragmatic biochemical thresholds such as lactate clearance, bile output, perfusate pH and glucose consumption [11, 19, 20, 36]. In this cohort, only hepatocellular criteria were systematically assessed, as all grafts originated from DBD donors for whom these metrics remain the accepted clinical standard [11, 20, 36]. Biliary parameters such as bile pH or bicarbonate, primarily validated in DCD grafts [18, 28, 37, 38] were not applied. Consistent with previous reports, conventional viability readouts did not differ between grafts with and without biliary complications, confirming that some grafts fulfilling these thresholds can still fail after transplantation [8, 10]. Although limited by sample size, this clinical observation aligns with our proteomic findings, which reveal early metabolic and stress‐response programmes undetectable by standard monitoring. Unlike dynamic bile chemistry, which primarily reflects short‐term secretory function during perfusion and has been mainly validated in DCD grafts, tissue proteomics captures underlying molecular stress responses, mitochondrial alterations, and inflammatory activation that may precede overt functional impairment. In DBD grafts, where biliary injury may not translate into immediate alterations in bile composition during NMP, this deeper molecular characterisation may provide complementary and earlier insight into graft susceptibility that is not captured by conventional biliary viability criteria [32].

Previous studies have focused mainly on bile or perfusate analyses [10, 13, 14, 24]. Bile proteomics during sequential perfusion has been shown to mirror peribiliary gland activity and cholangiocellular integrity, whereas perfusate analyses identified regenerative proteins but without correlation to outcomes [3]. Experimental multi‐omics studies in perfusion models have highlighted mitochondrial and inflammatory adaptations [37, 38]. However, to our knowledge, no study has systematically profiled the liver parenchyma itself during clinical NMP and directly correlated tissue proteome dynamics with biliary outcomes. By examining the parenchyma rather than secretions, our study delineates hepatocellular metabolic and proteostatic trajectories that converge with clinical evolution. Recent transcriptomic analyses have reported changes in immune, stress, and remodelling pathways during NMP [38] yet proteomics more accurately reflect the phenotypic state [39]. Direct association between liver tissue proteome and biliary outcomes has not previously been established in a clinical setting.

A central finding of this study is the early glycolytic activation in uncomplicated grafts, accompanied by relative attenuation of oxidative phosphorylation, evident at B2/B1 and persisting at B3/B2. In a glucose‐rich environment, this transient glycolytic shift may ensure rapid ATP resynthesis while limiting mitochondrial reactive oxygen species production, a pattern consistent with stress‐adaptive reprogramming described in ischemia‐tolerant hepatocytes [40]. In contrast, grafts that developed biliary complications lacked this glycolytic adaptation and instead showed modest OXPHOS upregulation, suggesting that metabolic flexibility rather than absolute mitochondrial activity may be a determinant of graft resilience.

At reperfusion (B3/B2), grafts with complications exhibited activation of translational machinery and enrichment of pathways related to ER stress and inflammation. Given the brief time window, this upregulation likely reflects a maladaptive stress response rather than productive protein synthesis [41, 42]. The enrichment of a hypoxia‐response signature in the uncomplicated group, associated with an increase of ELOC and proteasome subunits levels, further differentiated the two groups [35]. These observations suggest that proteasome‐mediated protein turnover may contribute to cellular resilience by sustaining proteostasis during reperfusion stress.

The reduced discrimination observed for the overall B3/B1 comparison likely reflects the integration of multiple overlapping biological processes occurring throughout procurement, perfusion, implantation and systemic reperfusion. While early intervals (B2/B1 and B3/B2) capture specific adaptive and stress responses, the B3/B1 ratio aggregates these sequential events into a composite signal, thereby attenuating group‐specific differences. These findings suggest that the biologically meaningful divergence between grafts occurs during early adaptive phases rather than in the cumulative endpoint measurement. Ischemic cholangiopathy is characterised by microvascular injury, oxidative stress and impaired cholangiocyte repair [43]. The absence of glycolytic activation, together with exaggerated translational and ER‐stress signalling may reduce the ability to buffer reperfusion injury, predisposing to biliary damage. While associations cannot infer causality, these data provide a mechanistic rationale for why conventional hepatocellular readouts fail to anticipate biliary complications. Conventional hepatocellular parameters primarily reflect global parenchymal metabolic recovery and do not specifically interrogate cholangiocyte integrity, peribiliary gland viability or microvascular injury within the biliary tree. Consequently, localised epithelial stress or mitochondrial dysfunction may remain undetected despite apparently satisfactory hepatocellular performance during perfusion [10].

From a translational perspective, integrating targeted markers of glycolytic activation and proteostasis capacity could complement current viability criteria. While mass spectrometry remains resource‐intensive, broad proteomic patterns could be converted into minimal, rapid assays suitable for end‐ischemic decision‐making, such as ambient mass spectrometry (SpiderMass) or focused immunoassays. Beyond assessment, conditioning strategies that promote glycolytic flux, enhance proteasome activity or temper maladaptive translational signaling during NMP merit evaluation as potential means to reinforce graft resilience. Such strategies could include pharmacological modulation of cellular metabolism during NMP or targeted interventions aimed at limiting oxidative and translational stress. These approaches are not yet used clinically because their impact on biliary outcomes and safety in human grafts remain insufficiently validated [44].

This study has limitations. It was conducted at a single centre with a modest cohort size, limiting statistical power and precluding multivariable adjustment. Biliary complications were analysed as a single outcome, although they represent a heterogeneous spectrum, including non‐anastomotic and anastomotic strictures as well as bile leaks that may result from distinct mechanisms such as ischemic injury, technical factors, or impaired cholangiocellular regeneration. Although early biliary leaks and anastomotic strictures may be influenced by technical factors, they may also depend on cholangiocyte viability, peribiliary microvascular integrity and epithelial repair capacity. We therefore considered leaks and strictures as part of a broader spectrum of biliary complications potentially reflecting underlying graft vulnerability rather than purely technical events. Importantly, our aim was not to attribute causality but to explore whether pre‐reperfusion molecular signatures correlate with clinically relevant biliary outcomes. We acknowledge that this heterogeneity may have attenuated specific molecular associations, and our findings should therefore be interpreted as reflecting an overall susceptibility to biliary injury rather than a specific lesion subtype. In addition, all grafts were DBD‐derived. Although the donor risk index values appeared relatively moderate, the majority of grafts fulfilled national extended criteria donor (ECD) definitions, reflecting current allocation practices in which ECD grafts represent approximately 80% of transplanted livers in our centre. Viability assessment relied solely on hepatocellular parameters; integrating cholangiocellular criteria and tissue proteomics in future studies will be an important next step. Despite these limitations, the consistency of metabolic and proteostatic signatures across time points supports the biological robustness of these findings. A prospective multicentre clinical trial (ClinicalTrials.gov identifier NCT06950398, TRANSPERF) has been initiated to assess the viability of discarded donor livers during normothermic machine perfusion. Within this trial, a translational sub‐study will extend the present proteomic work to prospectively validate molecular signatures of graft viability and biliary resilience.

In conclusion, tissue‐based proteomics during clinical NMP and early reperfusion uncovers distinct metabolic and proteostatic trajectories associated with post‐transplant biliary outcomes. The identification of glycolytic activation with preserved protein turnover in uncomplicated grafts, versus translational and ER‐stress programmes in complicated cases, provides a mechanistic framework for refining viability assessment beyond conventional biochemical thresholds. Validation in larger multicentre cohorts and translation into rapid targeted assays will be essential to integrate these insights into clinical practice.

## Author Contributions

H.J. conceived the study, performed NMP procedures, collected clinical data and drafted the manuscript. A.‐A.R., J.‐W.D. and C.D. performed the proteomic experiments and data analysis. H.P., E.B.N., S.T., B.T. and P.A. contributed to data acquisition and interpretation. K.B. and M.S. supervised the study. All authors critically revised the manuscript and approved the final version.

## Funding

This study was supported by internal institutional funding from Rennes University Hospital and the Inserm UMR_S 1085 (IRSET). The proteomic analyses were performed with the support of the TBM‐Core OncoProt platform (Bordeaux, France). No external commercial funding was received.

## Ethics Statement

This study was approved by the institutional ethics committee of Rennes University Hospital (Approval No. 25.139) and was conducted in accordance with French regulations governing research involving human biological material (MR‐004).

## Consent

Written informed consent was obtained from all participants in accordance with national regulations.

## Conflicts of Interest

The authors declare no conflicts of interest.

## Supporting information


**Appendix S1:** Procurement and machine perfusion settings.
**Table S1:** Viability criteria for determining liver viability at 4 h of NMP.
**Table S2:** Individual viability parameters of livers perfused at 4 h of NMP.
**Table S3:** Types and management of biliary complications in the NMP group.
**Table S4:** List of proteins significantly deregulated between the groups with and without complication.
**Table S5:** Detailed results of the Ingenuity Pathway Analysis of proteins that are significantly different between the two clinical groups with or without complications.

## Data Availability

The mass spectrometry proteomics data have been deposited to the ProteomeXchange Consortium via the PRIDE partner repository with the dataset identifier PXD067270. Additional data supporting the findings of this study are available from the corresponding author upon reasonable request.
